# Increasing Women’s Knowledge about HPV Using BERT Text Summarization: An Online Randomized Study

**DOI:** 10.3390/ijerph19138100

**Published:** 2022-07-01

**Authors:** Hind Bitar, Amal Babour, Fatema Nafa, Ohoud Alzamzami, Sarah Alismail

**Affiliations:** 1 Information Systems Department, King Abdulaziz University, P.O. Box 80200, Jeddah 21589, Saudi Arabia; ababor@kau.edu.sa; 2 Computer Science Department, Salem State University, Salem, MA 01970, USA; fnafa@salemstate.edu; 3 Computer Science Department, King Abdulaziz University, P.O. Box 80200, Jeddah 21589, Saudi Arabia; ualzamzami@kau.edu.sa; 4 Center for Information Systems and Technology, Claremont Graduate University, Claremont, CA 91711, USA; sarah.alismail@cgu.edu; 5 Beckman Research Institute, City of Hope, Duarte, CA 91010, USA

**Keywords:** BERT, cervical cancer, HPV, machine learning, text summarization

## Abstract

Despite the availability of online educational resources about human papillomavirus (HPV), many women around the world may be prevented from obtaining the necessary knowledge about HPV. One way to mitigate the lack of HPV knowledge is the use of auto-generated text summarization tools. This study compares the level of HPV knowledge between women who read an auto-generated summary of HPV made using the BERT deep learning model and women who read a long-form text of HPV. We randomly assigned 386 women to two conditions: half read an auto-generated summary text about HPV (*n* = 193) and half read an original text about HPV (*n* = 193). We administrated measures of HPV knowledge that consisted of 29 questions. As a result, women who read the original text were more likely to correctly answer two questions on the general HPV knowledge subscale than women who read the summarized text. For the HPV testing knowledge subscale, there was a statistically significant difference in favor of women who read the original text for only one question. The final subscale, HPV vaccination knowledge questions, did not significantly differ across groups. Using BERT for text summarization has shown promising effectiveness in increasing women’s knowledge and awareness about HPV while saving their time.

## 1. Introduction

Globally, cervical cancer, cancer that starts in the cervical area, is the fourth most common type of cancer in women [1]. Its prevalence is a global health crisis that causes a large number of female deaths annually [2]. The Cancer Genome Atlas Research Network reported that there were around 528,000 new cervical cancer cases worldwide in 2017, and almost 50% of those die yearly due to cervical cancer [2]. The leading cause of this gynecological cancer is the long-lasting and untreated Human Papillomavirus (HPV) infection. HPV is the cause of about 95% of total diagnosed cervical cancer cases [2].

Frequent screening tests and HPV vaccines can help prevent cervical cancer. Previous studies have shown the association between health literacy and cancer screening; increasing health literacy can help individuals to understand the severity of the disease and, subsequently, make them more likely to adopt health preventive behaviors [3,4]. Early HPV diagnosis can make it highly treatable and can help women survive cervical cancer and live a quality life. Nonetheless, in many countries, women’s awareness of, and knowledge about, HPV are limited and insufficient [1]. As reported by [1], the majority of women who participated in their focus groups did not know that HPV is the main reason for cervical cancer. Many studies focused on HPV and cervical cancer reported on the lack of knowledge about their deadly effects and called for more research to increase awareness and knowledge about HPV and cervical cancer [5,6,7,8]. In western China, only 28.85% of 1109 participants had heard about HPV [5]. Another study conducted among school-aged youth in the Bahamas showed that only 10.7% of 1364 sexually active participants had heard about HPV [6]. Thus, increasing awareness and knowledge about HPV could help to reduce cervical cancer cases and female deaths globally.

Attention ed: which research society? The research society must address the lack of awareness and knowledge about HPV, including in the areas of information systems and information technology. Most previous studies focusing on cervical cancer control in these two areas relied solely on promoting HPV vaccination using SMS, gamified web applications, or social media [8]. Despite the availability of many valuable resources for learning about HPV from a large number of trustworthy health and medical organizations such as the World Health Organization (WHO), the busy lifestyle of many women worldwide prevents them from gaining the required knowledge and being aware of the life-threatening and long-term effects of this virus.

One fundamental challenge to women’s ability to acquire the necessary knowledge and awareness of HPV is the large amount of information. Machine learning techniques, such as deep learning to reduce information overload and focusing on the most valuable information through text summarization, have been validated in previous studies [9]. To the best of our knowledge, deep learning has not yet been used in the context of increasing awareness of, and knowledge about, HPV. Therefore, in this paper we investigated the effect of using a deep learning summarization technique in increasing women’s knowledge about HPV. Information about HPV was aggregated from multiple sources for the sake of completeness and comprehensiveness. The Bidirectional Encoder Representations from Transformers (BERT) model, developed by Google, was utilized to generate a summary of the aggregated HPV information [10]. BERT is a bidirectional model that has been used to handle long-term dependencies. It relies on a self-attention mechanism, which determines the contextual relationship between words in text [11]. To evaluate the effectiveness of the BERT-summarized text, an online randomized study was undertaken, to assess the knowledge gained by two groups of women who read either the summarized, or the original, text. The results of this study provide the first evidence of the effectiveness of using deep learning-based text summarization to increase knowledge and awareness of women about HPV and cervical cancer. Results from this study could inform the usefulness of deep learning text summarization in educating learners while saving valuable time.

## 2. Literature Review

### 2.1. Deep Learning in the Medical Domain

Recently, several researchers have used deep learning and natural language processing (NLP) in the medical field [12,13,14,15,16,17,18,19,20,21,22]. Deep learning has been used to help physicians with the time and resource consuming task of real-time patient diagnosis. Chai et al. [21] utilized a deep learning technique to diagnose patients with glaucoma, an eye disease that affects and harms the eye’s health through the optic nerve and leads to vision loss. Deep learning models are also used to analyzed medical literature. Zhou and Li [18] designed a novel deep learning model to analyze Chinese medical literature and identify sections. The developed model considers the dependencies between sentences and the structure of articles to tackle the problem of section identification. In addition, deep learning has been used for real-time syndromic surveillance using social media texts linked to relevant news articles. Șerban et al. [19] proposed a model that has the potential to detect influenza-like symptoms, based on Twitter data, and display this information to raise situational awareness about disease spread. However, existing studies that use deep learning for the medical domain are still limited. In addtion, the performance of most deep learning models still has some drawbacks [23,24].

At the end of 2018, Google has built one such model, named BERT, that outperforms nearly all existing deep learning models in several NLP tasks [23,24,25]. BERT has recently obtained state-of-the-art results for a wide variety of NLP tasks, such as extracting clinical information for breast cancer [26] and analysis for biomedical clinical data [27]. Fan et al. [17] proposed using the BERT model for adverse drug events (ADEs) detection and extraction from online open data, since it allowed more drug side effects to be identified for doctors. Kalyan and Sangeetha [23] utilized the BERT model on a medical concept normalization system. The system maps health-related entities mentioned in social media free text such as symptoms, drugs, or adverse drug reactions into formal medical concepts. The BERT model was also used by [28] to extract Adverse Drug Events (ADEs) and their side effects from Food and Drug Administration (FDA) drug labels. Datta et al. [12] used the BERT model on a radiology report named Rad-SpRL to extract special information about patients, whereas, in [26], the model was used to extract comprehensive information covering clinical concepts and relations from a clinical report dataset written in the Chinese language. Likewise, the BERT model was used in [29] to extract tumor morphology from a dataset written in the Spanish language. Another application of BERT is the use of semantic features extraction for the detection of public emotion in social media posts. Blanco and Lourenço [20] used a BERT-based model to investigate the effectiveness of the automatic detection of optimistic and pessimistic feelings in 150,503 tweets about the COVID-19 health crisis. 

### 2.2. Text Summarization in the Medical Domain

The use of text summarization in the medical field has been reported in the literature [30]. Medical documents summarization has received significant research attention in different fields such as bioinformatics [31], imaging informatics [32], clinical informatics [26,27], and public health informatics [33,34,35]. Gayathri and Jaisankar [33] developed and applied a semantic dynamic summarizing technique to extract important sentences from medical documents. Moradi and Ghadiri [31] proposed a biomedical text summary method employing a concept-level analysis of text using itemset mining. Sarkar, Nasipuri [34] proposed a machine learning model with feature extraction to summarize articles. A graph-based method and MeSH descriptors were used by [35] to cluster medical articles into different groups and then summarize each group. Singh et al. [32] proposed a show, tell, and summarize model. Using an encoder–decoder framework, the model can generate findings from a given medical image. Using an encoder–decoder framework of neural summarization, the model summarizes the generated findings of the abnormalities, and the diseases appear in the medical image in a textual radiology report. The experiment results showed the effectiveness of their proposed model.

### 2.3. Research Gap and Research Objectives

The evaluation of text summarization techniques can be intrinsic or extrinsic. Using intrinsic evaluation, the quality of the summarized text is assessed based on metrics or attributes, such as ROUGE, accuracy, comprehensiveness, and relevancy [9,36]. The extrinsic evaluation assesses the summaries by applying them to user-related tasks, such as information seeking, to measure the user’s efficiency, comprehension, success rate, or decision-making accuracy [9,36]. It is concerned with evaluating how effective the automatically generated summaries are in helping users perform their normal tasks in realistic contexts to achieve their desired goals. Most of the evaluations of existing summarization techniques, including BERT, have used intrinsic evaluations, which focus on the components of the text summarization method and ignore the impact of text summarization in real-world settings [9,36,37]. However, for better maturity of the text summarization field in the medical and health informatics domain, more studies involving users need to be conducted. Such studies should focus on the cognitive aspects of users and evaluate the effect of text summarization on users’ performance and outcomes. To the best of our knowledge, none of the proposed work utilized deep learning, in general, or BERT, in particular, to summarize health education text about HPV from multiple sources with the objective of measuring the effectiveness of the summarized text in increasing women’s knowledge about this disease.

The goal of this research study is to determine the effect of using a deep learning text summarization technique to increase women’s knowledge of HPV. Thus, the main objective of this research article is to investigate whether the use of a deep learning summarization technique increases women’s knowledge about HPV. To do so, we compared the level of HPV knowledge between women who read an auto-generated summary of HPV made using the BERT deep learning model and women who read a long-form text about HPV.

## 3. Methods

### 3.1. Intervention

#### 3.1.1. Original Text

The original text was compiled from three different published articles to ensure that it contained the information required to answer all items on the HPV knowledge measure questionnaire [38,39,40]. No changes were made to the content of the source articles, nor were the sentences and their structure altered or updated. There was a total of 115 sentences in the original text (see Supplementary Materials).

#### 3.1.2. Auto-Generated Summarized Text

The BERT model was used to auto-generate a summary of 20 sentences from the original text comprising 115 sentences. The resultant summarized text was comprised of 20 sentences, which is approximately 17% of the original text. No changes were made to the content of the auto-generated summary nor were its sentences and their structures altered or updated (see Supplementary Materials).

### 3.2. Study Design

This study was approved by the Institutional Review Board (IRB) at Salem State University (IRB00006274). The study is an online randomized trial where participants were allocated into two groups. Group 1 read the summarized text while group 2 read the original text. The participants were recruited using the Amazon Mechanical Turk (MTurk) platform. MTurk participants are more attentive to instructions; therefore, MTurkers are suitable for recruitment in social science research [41]. Participants received $0.50 upon completion. The participants were informed that the survey task would take about 18 min, or 29 min based on their assigned group. The minimum time required for each group to complete the task was determined based on the rate of the learning process suggested by Carver’s model [42]. This model indicates that people can read 200 words within a minute if they read the text for learning purposes. In this study, the text presented to group 1 consisted of 422 words; it was estimated that the reading would take about 2.1 min to complete. The text shown to group 2 consisted of 2642 words; it was estimated that the reading would take about 13.2 min to complete. Approximately fifteen additional minutes were added for each group to complete the whole task. Further instructions were provided to participants in both groups, explaining that the time given was a minimum required time for reading the text. Participants were required to wait for that time to end before moving on to the post-test survey. However, participants who wished to have more time to finish reading the text were allowed to spend more time reading the text. This ensured that participants would spend enough time reading to learn the presented information about HPV.

#### 3.2.1. Eligibility and Randomization

**Figure 1:**
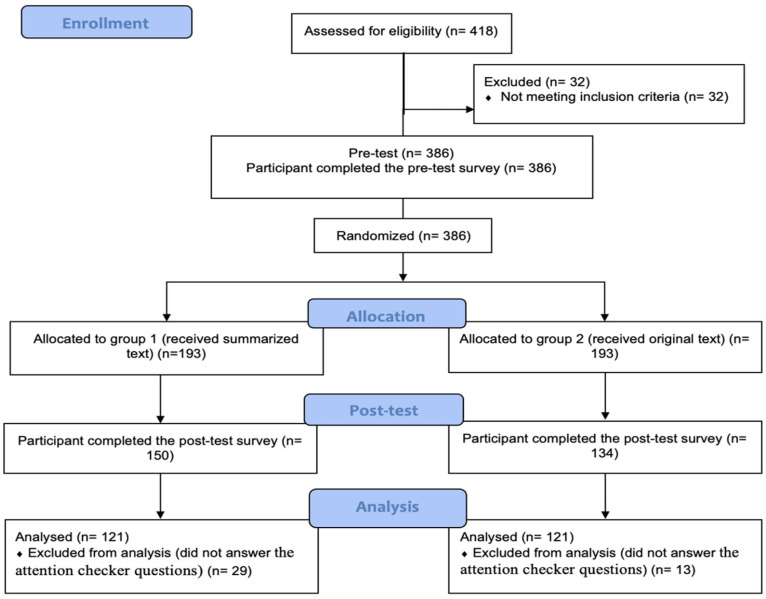
The flow diagram of the progress of the online randomized trial.

According to the Centers for Disease Control and Prevention (CDC), the age group for HPV-associated cancers in the US starts among women aged 20 and above [43]. Thus, participants in this study were women, aged 20 years old and above. Additionally, they were required to be able to speak, read, and write English fluently. Eligible women received an online consent form that described the purpose of the study. After they agreed to participate, women were then assigned to either group 1 or group 2 using block randomization to ensure each group has an equal sample size [44]. Block randomization is useful when participants are not available before starting the study and are recruited as the study is taking place. Blocks are used in small increments, which keeps the number of participants during the study balanced across the groups. In our study, each participant was randomly allocated to either the original text condition or the summarized text condition, with a ratio of 1:1, using Qualtrics’ block randomization and was not stratified. Figure 1 shows the flow diagram of the study.

#### 3.2.2. HPV Knowledge Measurement

We measured the participants’ pre- and post-knowledge using the US version of Waller et al.’s (2013) HPV knowledge measure. The validity and reliability of the US version of the HPV knowledge measure are described and reported elsewhere [45]. In the baseline assessment, participants were asked if they had heard of HPV. Those who answered “yes” responded to 16 items that measure HPV knowledge. Next, they were asked if they have heard of HPV testing and if so, they responded to six items that measure HPV testing knowledge. They were then asked if they had heard of HPV vaccination. Those who answered “yes” were asked to answer seven items that measure HPV vaccination knowledge. This yielded 29 items for the total HPV knowledge. The response options for all items were true, false, and do not know.

#### 3.2.3. Participant’s Recruitment

The study advertisement was placed on the MTurk platform. The study description and eligibility screening were presented on a separate survey that is linked to the MTurk portal, which was created in Qualtrics. A brief description of the tasks, the estimated time needed for completion, and the monetary reward upon completing the task were clarified to the participants before beginning the tasks. Women who were willing to participate in this study clicked on a link that directed them to complete a screening questionnaire to determine their eligibility (women must be 20 years or older and can speak, read, and write English fluently). Those who were eligible were sent to an online consent form that described the purpose of the study. After signing the consent form, a pre-test survey was administered to the participants to determine their pre-existing knowledge about HPV. Participants were then assigned randomly to one of the two groups (group 1 or group 2). After filling out the pre-test survey, women in group 1 read the summarized text while women in group 2 read the original text. Next, women in both groups completed the post-test survey. To ensure that participants focused and paid attention to the task, two attention checks were added in each pre and post surveys. The attention check questions were: (1) choose “False” as an answer in the following question, and (2) choose “True” as an answer in the following question. Responses were also reviewed manually to verify the completeness of the questionnaires.

### 3.3. Statistical Analysis

Statistical analyses were performed using IBM Statistical Package of Social Sciences software (SPSS; version 26.0, SPSS Inc., Chicago, IL, USA). Descriptive statistics were computed using frequencies, percentages were used for categorical variables, and means and standard deviations were calculated for continuous variables. Normality of data was checked using Kolmogorov—Smirnov test. The scores of the HPV knowledge related outcomes were normally distributed for both groups, and thus, data were presented as means (SD). The assumption of homogeneity of variances was tested using Levene’s test for equality of variances. The test showed that the variances of the general HPV knowledge, HPV testing, and HPV vaccination in the two groups were not statistically significant (F (1,240) = 0.06, P = 0.81; F (1,240) = 0.08, P = 0.78; F (1,240) = 2.6; P = 0.12, respectively). Thus, this assumption is met.

The reliability scores of the three HPV knowledge subscales (i.e., general HPV knowledge, HPV testing, and HPV vaccination) were evaluated using Kuder—Richardson Formula 20 (KR-20). Cross tabulation and Chi-square statistical tests were performed to compare the proportions of correct responses for each item between the two groups. An independent *t*-test was used to compare the mean scores of each HPV knowledge subscale and the total HPV knowledge score between groups 1 and 2 after the intervention. For those participants who reported having heard about HPV, HPV testing, and HPV vaccination at baseline, a general linear model was employed to compare mean scores of the total HPV knowledge after the intervention between the two groups with adjustment for the baseline scores. A *p*-value of <0.05 was considered statistically significant.

The main outcome variables were general HPV knowledge, HPV testing knowledge, HPV vaccination knowledge, and total HPV knowledge. The scores for the first three variables were calculated. A score of 1 was given for a correct answer/response and a score of zero for an incorrect or “I do not know” response. The sum of the responses’ scores was calculated in each corresponding subscale, with a final score ranging from 0 to 16 for general HPV knowledge, from 0 to 6 for HPV testing knowledge, and from 0 to 7 for HPV vaccination knowledge. A total HPV knowledge score was then calculated by summing the scores of responses in each subscale; scores ranged from 0 to 29, with lower scores indicating poor knowledge.

## 4. Results

A total of 418 women were recruited to participate in this study. A total of 176 were excluded due to their incomplete responses (32 women didn’t meet the inclusion criteria, 102 women didn’t complete the post-test survey, and 42 women didn’t answer the attention checker questions). Thus, 242 responses were available for further analysis. The baseline characteristics of the participants are displayed in Table 1. The mean age of participants was 37.8 years (SD = 12.2), the majority were Caucasian (76.8%), and had a bachelor’s degree (40.5%). The KR-20 of the general HPV knowledge was found to be 0.845 (above 0.70) which is considered a high level of internal consistency. At baseline, no statistically significant differences were found between groups 1 and 2 regarding women reporting having heard of HPV, having heard of HPV testing, and having heard of HPV vaccination (*p* > 0.05; see Table 2).

Comparisons between groups 1 and 2 according to participants’ answers to each item of the three subscales (general HPV knowledge, HPV testing knowledge, and HPV vaccination knowledge) after receiving the intervention are illustrated in Table 3, Table 4 and Table 5.

Regarding the general HPV knowledge (shown in Table 3), there was a statistically significant difference between group 1 and group 2 answering the questions “There are many types of HPV” and “HPV usually does not need any treatment” (*p* < 0.05). Group 1 gave significantly fewer correct answers than group 2 (81% vs. 92%) for the question about the HPV types. In contrast, group 2 gave significantly fewer correct answers than group 1 (38.8% vs. 68.6%) for the question concerning the necessity of treating HPV. There was no statistically significant difference between group 1 and group 2 for the remaining questions (*p* > 0.05). However, the rate of giving correct answers in the following questions: “HPV can cause cervical cancer”, “HPV can be passed on during sexual intercourse”, “Having sex at an early age increases the risk of getting HPV”, and “Most sexually active people will get HPV at some point in their lives” in group 1 were slightly higher than group 2 (96.7% vs. 92.6%), (94.2% vs. 91.7%), (90.9% vs. 86%), and (64.5% vs. 62.8%), respectively.

Looking at the HPV testing knowledge questions presented in Table 4, the result shows that there is a statistically significant difference in the proportion of women who gave correct answers to only one question (i.e., when you have an HPV test, you get the results the same day) (*p* < 0.05), with group 1 being slightly higher than group 2 (34.7% vs. 21.5%). Regarding the HPV vaccination knowledge items, shown in Table 5, there were no statistically significant difference between the two groups (*p* > 0.05).

A comparison of results of the three subscales and total HPV knowledge is summarized in Table 6. For the HPV testing knowledge, there was a statistically significant difference between group 1 and group 2 (*p* < 0.05). There were no statistically significant differences in the mean scores between the two groups post the intervention on the general HPV knowledge subscale, HPV vaccination subscale, or total HPV knowledge (*p* > 0.05).

When we examined the total HPV knowledge after the intervention, adjusted for total HPV knowledge at baseline, for those who have heard of HPV, HPV testing, and HPV vaccination at baseline (*N* = 172), the mean score of the HPV knowledge were 22.07 and 22.43 for Groups 1 and 2, respectively. As shown in Table 7, there were no statistically significant differences in post-intervention total HPV knowledge between the two groups when adjusted for pre-intervention total HPV knowledge (F = 0.591, *p* = 0.443). It is important to note that all ANCOVA assumptions (normality and homogeneity) were checked before we run the analysis as described in the Statistical Analysis section.

## 5. Discussion

HPV is the most common sexually transmitted infection that causes cervical cancer. It contributes to 17,600 known cases of cervical cancer in women annually [46,47]. While HPV vaccination, early detection, and treatment of this virus can help women prevent its long-term and life-threatening effects, few women have the necessary knowledge and awareness to do so. Despite the availability of online educational resources about HPV, busy lifestyles and competing priorities may prevent many women around the world from obtaining the necessary knowledge about HPV. Thus, there is an essential need to educate women about HPV. One tool that can be used to mitigate the lack of HPV knowledge is auto-generated text summarization of educational materials. Text summarization techniques can extract the most informative text from a long document about HPV to deliver concise and relevant information to the readers. In this study, a deep learning text summarization technique was utilized to evaluate whether an auto-generated summary about HPV increased women’s knowledge about HPV. This is the first online randomized study that uses a deep learning summarization technique to increase women’s knowledge about HPV. This study evaluates the summarization system in a simulated real-world setting with representative users. Unlike most of the previous studies focusing on intrinsic evaluation [9,36,37], this study focuses on extrinsic evaluation, which could assess the impact it has on patients’ knowledge and outcomes. The evaluation used in this study could provide directions for the future, to improve the summarization of medical texts to help real users achieve their goals.

In recent years, NLP and deep-learning approaches have been widely adopted in health care fields to improve human health. Multiple advanced techniques are now available to help machines analyze open medical texts. Previous text analysis research in the medical field has addressed finding information related to health care in medical texts [18,21], identifying drug information [17], classifying social media text into health-related problems or different levels of severity for these problems [19,20,22], and summarizing medical articles [31,32,34,35]. However, none of the previous studies have applied deep learning techniques on medical health articles for increasing women’s knowledge about HPV and its associated infections. To the best of the authors’ knowledge, this is the first online randomized study that uses a deep learning summarization technique, specifically the BERT model, for the purpose of increasing public health knowledge amongst members of the general public.

### 5.1. Principal Findings

The purpose of this study was to compare the effectiveness of a deep learning summarization tool across a validated 29-item questionnaire that was divided into three sub-scales: general HPV-, HPV testing-, and HPV vaccination-knowledge. The questionnaire was completed by MTurk workers who had read either an original article about HPV and cervical cancer or an auto-summarized version of the article produced by a BERT model.

For the general HPV knowledge sub-scale responses, the women who were given the original text correctly answered more general knowledge questions about HPV than those who were given the summarized text. Specifically, those who read the original text were more likely to correctly answer the following two questions: (1) There are many types of HPV, and (2) HPV usually does not need any treatment. However, this discrepancy may be due to the relevant information required to answer these two questions not being addressed clearly in the auto-generated shortened summary. The level of summarization in our study was adjusted to 17% of the original text, which might have affected whether adequate details were present in the summary to answer these two questions. This suggests that human judgment might be needed to ensure the completeness and the comprehensiveness of the summarized text. The summarized text may present a way to increase women’s knowledge about their own personal health, as long as human judgment is used to ensure completeness of information.

When the HPV testing knowledge subscale responses were analyzed, those who read the original text were more accurate for only one question: when you have an HPV test, you get the results the same day: (true, false, do not know). They were more likely to correctly answer “false” than those who read the auto-generated summary. The fact that only one question in the subscale differed between groups indicates that the use of auto generated summaries is likely effective. To further support this interpretation, the final subscale (HPV vaccination knowledge questions) did not differ as a function of what text was read.

### 5.2. Empirical Implications

This work has implications for practical applications, including the use of auto-summarization tools for public health education material development, and the uses and limitations of the BERT model in the context of auto-summarizing health education articles. Summarizing health education articles using a deep learning approach is of added value for the development of health educational materials that support public health. Studies have shown that information overload can increase mental effort and be fatal when it hinders understanding medical and health-related information [30]. The usage of the BERT model in text summarization reduced the length of the document while preserving the essence of the document, as reported in earlier studies [23,26,28]. Additionally, our results showed that, despite the simplification of the auto-generated summarized text to save the readers time and effort, the knowledge gained by those who read the summarized text was comparable to those who read the original text. In other words, the similarity of the results between groups indicates that the summarized text was sufficient, or just as informative, as the original text and had the advantage of taking less time to read. The use of auto-generated summaries, while useful in many cases, did lead to some errors. For their future use, we would encourage a thorough review of all summarized text prior to distribution to ensure that accuracy, relevancy, and face validity requirements are met.

The features of the BERT model are promising for those who are interested in using machine learning to increase public knowledge of various health conditions, above and beyond HPV. The BERT model, and the auto-generated summaries it produces, has promising implications to create educational materials for health interventions. In building different educational materials, it is useful to have a tool that can produce an auto-summary instead of having to manually summarize the text.

### 5.3. Limitations and Future Directions

The study has several limitations. It was an online study hosted on the MTurk platform, therefore only accessible to registered workers with an MTurk account. Moreover, the sample size slightly underpowered and may not have been able to detect statistically significant differences for small effects. Despite the limitations of this study, auto-generated summarization is a promising tool to increasing public health knowledge on various diseases and developing future health interventions.

Future studies should address two primary issues. First, different levels of text summarization should be investigated to determine the most effective and informative percentage of summarization. Second, the current study should be expanded to investigate the effectiveness of text summarization techniques in different contexts to provide further insights regarding the possible effects of using auto-generated summaries to gain knowledge and awareness. In addition, future studies could be directed towards integrating automatic text summarization with educational interventions and health behavioral changing models, which have been shown to be effective in cervical cancer prevention [48]. The Precede–Proceed model is an example of such models, which provides a comprehensive and well-structured framework for designing, implementing, and evaluating health promotion, public health, and disease prevention programs [49]. This model has been utilized effectively in designing educational interventions for raising awareness [48]. By integrating automatic text summarization with such a model, reassurance of behavioral and environmental changes can be obtained.

## 6. Conclusions

Increasing women’s awareness and knowledge of HPV is a healthcare priority since many women globally suffer from this infection and its associated life-threatening diseases, such as cervical cancer. One way to achieve the goal of increasing women’s awareness and knowledge of HPV is the use of deep learning text summarization techniques, such as BERT. These techniques are used to summarize related medical articles to reduce the amount of text, and consequently, the time needed to read it. Our findings showed the efficiency of using auto-generated summaries in increasing women’s knowledge and awareness. More research must be done to investigate the effect of applying different machine learning algorithms and techniques in health education.

## Figures and Tables

**Table 1:** Participants’ characteristics.

Characteristics	Group 1 (*N* = 121)	Group 2 (*N* = 121)	Total (*N* = 242)
**Age in Years**			
Mean (SD)	38.3 (12.8)	37.4 (11.6)	37.8 (12.2)
**Ethnicity, *n* (%)**			
Caucasian	94 (77.7)	92 (76.0)	186 (76.8)
Hispanic	7 (5.8)	12 (9.9)	20 (8.2)
American Indian	2 (0.8)	2 (1.7)	5 (2.1)
Asian	4 (1.6)	7 (5.8)	11 (4.5)
African	6 (2.5)	7 (5.8)	15 (6.2)
Other	8 (3.3)	1 (0.8)	5 (2.1)
**Highest education level, *n* (%)**			
High school	9 (7.4)	11 (9.1)	20 (8.2)
Associate degree	15 (12.4)	14 (11.6)	29 (11.9)
Some collage, no degree	22 (18.2)	24 (19.8)	46 (19)
Bachelor	50 (41.3)	48 (39.6)	98 (40.5)
Master	22 (18.2)	22 (18.2)	44 (18.2)
Doctorate	3 (2.5)	2 (1.7)	5 (2.1)

Group 1 = received the summarized text version; Group 2 = received the original text version.

**Table 2:** General HPV knowledge and awareness at baseline.

Items	Answers	Group 1	Group 2	Total	*p*-Value ^a^
**Before Today, Had You Ever Heard of HPV?**	Yes	112 (92.6)	116 (95.9)	228 (94.2)	0.449
	No	8 (6.6)	4 (3.3)	12 (5.0)	
	Don’t know	1 (0.8)	1 (0.8)	2 (0.8)	
**Have You Ever Heard of HPV Testing?**	Yes	94 (77.7)	90 (74.4)	184 (76.0)	0.698
	No	20 (16.5)	25 (20.7)	45 (18.6)	
	Don’t know	7 (5.8)	6 (5.0)	13 (5.4)	
**Before Today, Had You Ever Heard of HPV Vaccination?**	Yes	97 (80.2)	103 (85.1)	184 (76.0)	0.449
	No	21 (17.4)	17 (14.0)	45 (18.6)	
	Don’t know	3 (2.5)	1 (0.8)	13 (5.4)	

HPV = human papillomavirus. Group 1 = received the summarized text version; Group 2 = received the original text version. ^a^ Chi-square test.

**Table 3:** Comparison of general HPV knowledge after receiving the intervention between the two groups.

Items ^a^	Group 1			Group 2			*p*-Value ^b^
	Correct *n* (%)	Incorrect *n* (%)	Do Not Know	Correct *n* (%)	Incorrect *n* (%)	Do Not Know	
**Having Many Sexual Partners Increases the Risk of Getting HPV (T)**	114 (94.2)	4 (3.3)	3 (2.5)	117 (96.7)	3 (2.5)	1 (0.8)	0.335
**A Person Could Have HPV for Many Years Without Knowing It (T)**	104 (86.0)	14 (11.6)	3 (2.5)	108 (89.3)	12 (9.9)	1 (0.8)	0.435
**HPV Is Very Rare (F)**	99 (81.8)	18 (14.9)	4 (3.3)	93 (76.9)	25 (20.7)	3 (2.5)	0.341
**HPV Can Cause Cervical Cancer (T)**	117 (96.7)	3 (2.5)	1 (0.8)	112 (92.6)	9 (7.4)	0 (0)	0.154
**HPV Can Be Passed on During Sexual Intercourse (T)**	114 (94.2)	5 (4.1)	2 (1.7)	111 (91.7)	6 (5.0)	4 (3.3)	0.450
**HPV Always Has Visible Signs or Symptoms (F)**	88 (72.7)	26 (21.5)	7 (5.8)	95 (78.5)	26 (21.5)	0 (0)	0.295
**Using Condoms Reduces the Risk of Getting HPV (T)**	97 (80.2)	12 (9.9)	12 (9.9)	105 (86.8)	15 (12.4)	1 (0.8)	0.166
**HPV Can Cause HIV/Aids (F)**	83 (68.6)	28 (23.1)	10 (8.3)	83 (68.6)	31 (25.6)	7 (5.8)	1.00
**HPV Can Be Passed on by Genital Skin-to-Skin Contact (T)**	98 (81.0)	13 (10.7)	2 (1.7)	109 (90.1)	11 (9.1)	1 (0.8)	0.061
**Men cannot get HPV(F)**	101 (83.5)	15 (12.4)	5 (4.1)	98 (81.0)	19 (15.7)	4 (3.3)	0.614
**Having Sex at an Early Age Increases the Risk of Getting HPV(T)**	110 (90.9)	9 (7.4)	2 (1.7)	104 (86.0)	16 (13.2)	1 (0.8)	0.228
**There Are Many Types of HPV (T)**	98 (81.0)	13 (10.7)	10 (8.3)	112 (92.6)	5 (4.1)	4 (3.3)	0.008
**HPV Can Cause Genital Warts (T)**	108 (89.3)	9 (7.4)	4 (3.3)	108 (89.3)	11 (9.1)	2 (1.7)	1.000
**HPV Can Be Cured with Antibiotics(F)**	79 (65.3)	23 (19.0)	19 (15.7)	80 (66.1)	32 (26.4)	9 (7.4)	0.892
**Most Sexually Active People Will Get HPV at Some Point in Their Lives (T)**	78 (64.5)	32 (26.4)	11 (9.1)	76 (62.8)	35 (28.9)	10 (8.3)	0.789
**HPV Usually Doesn’t Need Any Treatment (F)**	83(68.6)	29 (24.0)	9 (7.4)	47 (38.8)	70 (57.9)	4 (3.3)	0.0001

Note: ^a^ Not all the 242 women answered the questions. ^b^ Chi-square test; T: Correct = 1, incorrect/don’t know = 0; F: Correct/don’t know = 0, incorrect = 1. Group 1 = received the summarized text version; Group 2 = received the original text version.

**Table 4:** Comparison of HPV testing knowledge after receiving the intervention between the two groups.

Items ^a^	Group 1			Group 2			*p*-Value ^b^
	Correct *n* (%)	Incorrect *n* (%)	Do Not Know	Correct *n* (%)	Incorrect *n* (%)	Do Not Know	
**If a Woman Tests Positive for HPV She Will Definitely Get Cervical Cancer (F)**	90 (74.4)	25 (20.7)	6 (5)	90 (74.4)	30 (24.8)	1 (0.8)	1.000
**An HPV Test Can Be Done at the Same Time as a Pap Test (T)**	101 (83.5)	8 (6.6)	12 (9.9)	103 (85.1)	13 (10.7)	5 (4.1)	0.724
**An HPV Test Can Tell You How Long You Have Had an HPV Infection (F)**	63 (52.1)	40 (33.1)	18 (14.9)	69 (57)	40 (33.1)	12 (9.9)	0.439
**HPV Testing Is Used to Indicate if The HPV Vaccine Is Needed (F)**	66 (54.5)	44 (36.4)	11 (9.1)	75 (62)	41 (33.9)	5 (4.1)	0.241
**When You Have an HPV Test, You Get the Results the Same Day (F)**	47 (38.8)	42 (34.7)	32 (26.4)	75 (62)	26 (21.5)	20 (16.5)	0.0001
**If an HPV Test Shows That a Woman Does Not Have HPV, Her Risk of Cervical Cancer Is Low (T)**	53 (43.8)	51 (42.1)	17 (14)	66 (54.5)	48 (39.7)	7 (5.8)	0.095

Note: ^a^ Not all the 242 women answered the questions. ^b^ Chi-square test; T: Correct = 1, incorrect/don’t know = 0; F: Correct/don’t know = 0, incorrect = 1. Group 1 = received the summarized text version; Group 2 = received the original text version.

**Table 5:** Comparison of HPV vaccination knowledge after receiving the intervention between the two groups.

Items ^a^	Group 1			Group 2			*p*-Value ^b^
	Correct *n* (%)	Incorrect *n* (%)	Do not know	Correct *n* (%)	Incorrect *n* (%)	Do Not Know	
**Girls Who Have Had an HPV Vaccine Do Not Need a Pap Test When They are Older (F)**	97 (80.2)	17 (14)	7 (5.8)	92 (76)	28 (23.1)	1 (0.8)	0.437
**One of the HPV Vaccines Offers Protection Against Genital Warts (T)**	86 (71.1)	24 (19.8)	11 (9.1)	98 (81)	17 (14)	6 (5)	0.071
**The HPV Vaccines Offer Protection against All Sexually Transmitted Infections (F)**	91 (75.2)	28 (23.1)	2 (1.7)	83 (68.6)	38 (31.4)	0 (0)	0.253
**Someone Who Has an HPV Vaccine Cannot Develop Cervical Cancer (F)**	93 (76.9)	22 (18.2)	6 (5)	92 (76)	25 (20.7)	4 (3.3)	0.880
**HPV Vaccines Offer Protection against Most Cervical Cancers (T)**	97 (80.2)	15 (12.4)	9 (7.4)	99 (81.8)	18 (14.9)	4 (3.3)	0.743
**The HPV Vaccine Requires Three Doses (T)**	69 (57)	23 (19)	29 (24)	79 (65.3)	30 (24.8)	12(9.9)	0.187
**The HPV Vaccines are Most Effective if Given to People Who Have Never Had Sex (T)**	91 (75.2)	17 (14)	13 (10.7)	94 (77.7)	19 (15.7)	8 (6.6)	0.649

Note: ^a^ Not all the 242 women answered the questions. ^b^ Chi-square test; T: Correct = 1, incorrect/don’t know = 0; F: Correct/don’t know = 0, incorrect = 1. Group 1 = received the summarized text version; Group 2 = received the original text version. HPV = human papillomavirus.

**Table 6:** Independent sample *t*-test between groups 1 and 2.

Item	Group	*N*	Mean (SD)	*p*-Value ^a^
**General HPV Knowledge**	1	121	12.97 (2.69)	0.282
	2	121	12.87 (2.76)	
**HPV Testing Knowledge**	1	121	3.47 (1.52)	0.017
	2	121	3.95 (1.58)	
**HPV Vaccine Knowledge**	1	121	5.15 (1.42)	0.586
	2	121	5.26 (1.63)	
**Total HPV Knowledge**	1	121	21.6 (4.74)	0.449
	2	121	22.09 (5.24)	

Note: HPV = human papillomavirus. T: Correct = 1, incorrect/don’t know = 0; F: Correct/don’t know = 0, incorrect = 1. ^a^ independent sample *t*-test. Group 1 = received the summarized text version; Group 2 = received the original text version.

**Table 7:** ANCOVA Comparison of total HPV knowledge after the intervention between the two groups with adjustment for the baseline scores.

Items	Mean ^a^		Std. Error		F	*p*-Value
	Group 1 (*N* = 83)	Group 2 (*N* = 89)	Group 1 (*N* = 83)	Group 2 (*N* = 89)		
**Total HPV Knowledge**	22.07	22.43	0.333	0.322	0.591	0.443

Note: HPV = human papillomavirus; Group 1 = received the summarized text version; Group 2 = received the original text version. ^a^ Adjusted for baseline score. Not all the 242 women answered the baseline questions.

## Data Availability

Not applicable.

## Supplementary Materials

The following supporting information can be downloaded at: https://www.mdpi.com/article/10.3390/ijerph19138100/s1.
