# Supplementary material for: Increasing Women’s Knowledge about HPV Using BERT Text Summarization: An Online Randomized Study
Source: Int J Environ Res Public Health. 2022 Jul 1;19(13):8100. doi: 10.3390/ijerph19138100 (PMC9265758; doi:10.3390/ijerph19138100)
Supplement: Supplementary file 1 [file ijerph-19-08100-s001.zip › ijerph-1739455-supplementary.pdf]

The original text and auto-generated summarized text

HPV and Cervical Cancer

Background

HPV is the most common viral infection of the reproductive tract and is the cause of a range of conditions in both men and women, including precancerous lesions that may progress to cancer. Although the majority of HPV infections do not cause symptoms and resolve spontaneously, persistent infection with HPV may result in disease. In women, persistent infection with specific HPV types (most frequently HPV-16 and HPV-18) may lead to precancerous lesions which, if untreated, may progress to cervical cancer. HPV infection is also associated with oropharyngeal and anogenital cancers and other conditions in men and women [2].

HPV is transmitted sexually, and certain sexual practices increase the risk for developing cervical cancer. The more sexual partners a woman has or the younger she begins sexual activity the greater the risk of acquiring a sexually transmitted disease such as HPV. The younger a woman becomes sexually active, the more likely she will be exposed to the virus. Infection with HPV is common, with 10%– 15% of the sexually active population between the ages of 18–28 infected. Only 1% of this population shows evidence of genital warts, and approximately 4% have abnormal cervical cytology [1]. Condoms can reduce but not eliminate the risk of HPV transmission, but because they are effective with other STDs, their use should be highly encouraged [1].

HPV Disease

HPV viruses are spread through contact with infected genital skin, mucous membranes, or bodily fluids, and can be transmitted through sexual intercourse including oral sex. Most (70–90%) of HPV infections are asymptomatic and resolve spontaneously within 1–2 years [2]. Most individuals who get an HPV infection never know they have it because symptoms often do not develop [1]. If not detected and treated appropriately, persistent infection with high-risk types may progress to invasive carcinoma at the site of infection, mainly of the genital tract. Persistent HPV infection is a necessary cause of cervical cancer [2].

Persistent HPV infection is defined by the presence of type-specific HPV DNA on repeated clinical biological samples over a period of time, usually 6 months, although this time period is not universally accepted. About 5–10% of all infected women develop persistent infection. Persistent infections, within months or years, may progress towards premalignant glandular or squamous intra-epithelial lesions, classified histopathologically as cervical intra-epithelial neoplasia (CIN), and to cancer. CIN is further classified as: CIN 1: mild dysplasia; CIN 2: moderate to marked dysplasia; and CIN 3: severe dysplasia to carcinoma in situ. Most CIN lesions regress spontaneously, though over a number of years, lesions on the cervix can slowly become cancerous [2].

The interval between the acquisition of HPV infection and progression to invasive carcinoma is usually 20 years or longer. The basis for this progression is not well understood but the predisposing conditions and risk factors include the following: HPV type; immune status (susceptibility is greater in persons who are immunocompromised, HIV-infected, or receiving immunosuppressive therapy); co-infection with other STIs (herpes simplex, chlamydia and gonococcal infections); parity and young age at first pregnancy; tobacco smoking. HIV-infected women have a higher prevalence of persistent HPV infection, often with multiple HPV types, and are at increased risk of progression to high- grade CIN and cervical cancer compared to women without HIV infection [2].

HPV infection is also implicated in a variable range of carcinomas of the anus (88%), the vulva (15–48%, depending on age) and vagina (78%), the penis (51%) and the oropharynx (13–60%, depending on region). In all of these sites HPV-16 is the predominant type [2].

HPV infection with low-risk types causes anogenital warts in females and males (condylomata acuminata or venereal warts). Over 90% of these are associated with types 6 and 11. The reported median time between infection with HPV types 6 or 11 and the development of anogenital warts is 11–12 months in men and 5–6 months in young women. Anogenital warts can be difficult to treat and, in rare cases, can become malignant [2].

Genital HPV infection is primarily transmitted by genital skin-to-skin contact, usually but not necessarily during sexual intercourse. HPV infection can occur at any age and has been re- ported in healthy young children. In a cross-sectional study of nearly 20 000 women aged 15–74 years without cervical lesions, age-standardized HPV prevalence varied more than 10-fold between populations. There is an in- verse relationship between age and hu- man papillomavirus (HPV) prevalence in many countries, but in some of the poorest areas studied HPV prevalence was high across all age groups. In some countries, cross-sectional and cohort studies have shown a U-shaped curve with a first peak in women under 30 years of age and a second peak in women aged 55–64 years [3].

Among women infected with HIV, a recent meta-analysis found that almost 40% of those with no cervical cytological abnormalities had HPV infection. Simultaneous infection with multiple HPV genotypes is more common in HIV-infected women than in women without HIV. HIV-infected men and women are at increased risk of HPV- associated anal cancer. HPV infection risk is associated with the number of sex partners that the woman or her partner has had over a lifetime and recently. Although some cross-sectional studies found no evidence of a reduction in HPV prevalence through condom use, lower HPV prevalence has been reported among women using condoms with their regular partners and a

longitudinal study found that consistent condom use protected American college students significantly against new HPV infections and appeared to protect against CIN lesion development [3].

Diagnosis of cervical HPV infection/disease

Several methods have been developed to screen for HPV. The accuracy of cervical cytology is responsible for a 70% decrease in cervical cancer mortality over the past 50 years and is one of the most effective screening tests at present. Conventional cervical cytology using a slide and fixative for a Pap test will detect premalignant lesions but not an HPV infection. The new liquid-based methods (versus the slide method) allow for not only the detection of premalignant conditions but also HPV. Liquid hybridization, Hybrid Capture® (Digene, Silver Springs, MD), or polymerase chain reaction methods can detect HPV in cervical samples and tissues. These are nucleic acid-based tests that can detect and type HPV. HPV screening is becoming more integrated into the care of women and is per- formed when an abnormality is detected by Pap test. Pap test results can fall into six major categories: normal and five abnormal categories [1]. A normal result is the most frequent (90%–95%). A result of atypical squamous cells of undetermined significance (ASCUS) indicates that squamous cells are detected and do not look entirely normal but also are not entirely abnormal. Approximately 60% of the women with this result are HPV negative with no cervical disease and 40% are HPV positive with detectable cervical changes, mostly low-grade type changes. A low-grade squamous intraepithelial lesion (LSIL) result is typically a result of an HPV infection in younger women (younger than 35) and in older women because of declining estrogen levels and other effects of the aging process on squamous cells. An estimated 75% of women with this result will test positive for HPV (ARHP). About 90% of women with a high-grade squamous intraepithelial lesion (HSIL) result will have cell changes because of HPV, the majority with high-grade cervical changes (ARHP). The atypical glandular cells of undetermined significance (AGCUS) indicate that glandular cells are detected and do not look entirely normal but also are not entirely abnormal. Upon further testing of

women with AGCUS, approximately 50% will be found to have a normal histology (ARHP). However, high-grade squamous and glandular lesions may be found in 20%–50% of women with this result (ARHP). The fifth category is cancer (squamous or adenocarcinoma). Rarely will a Pap test result be cancer in women obtaining regular Pap tests because cervical cancer is a slow-growing disease and precursor changes will be identified first (ARHP). When a woman goes years between Pap tests, a precursor lesion may be missed [1].

Treatment

Although there is no virus-specific treatment for HPV infection, screening and treatment for pre-invasive disease of the cervix is highly successful in preventing progression to cervical cancer. Cervical pre-cancerous lesions can be treated by ablative methods which include destruction of abnormal tissue by burning or freezing (cryotherapy) and surgical removal of abnormal tissue (loop electrosurgical excision procedure [LEEP] or cone biopsy). In low-income countries, where cervical cancer prevention and control exists, pre-cancerous lesions of the cervix are most commonly treated by cryotherapy. Surgical excision of the affected tissue is also effective (loop electrosurgical excision procedure) and necessary when the lesion is large. Excision by cone biopsy is reserved for more advanced or recurrent cases, especially those involving disease in the endocervical canal [2].

Vaccines

Three prophylactic HPV vaccines, directed against high- risk HPV types, are currently available and marketed in many countries worldwide for the prevention of HPV- related disease: the quadrivalent vaccine was first licensed in 2006, the bivalent vaccine in 2007 and the nonavalent vaccine in 2014. All of these vaccines are intended to be administered if possible before the onset of sexual activity, i.e. before first exposure to HPV infection. Using recombinant DNA technology, all 3 vaccines are prepared from the

purified L1 structural protein that self-assemble to form HPV type-specific empty shells, termed virus-like particles (VLPs). None of the vaccines contains live biological products or viral DNA, and are therefore non-infectious; they do not contain antibiotics or preservative agents [2].

By 31 March 2017, globally 71 countries (37%) had introduced HPV vaccine in their national immunization programme for girls, and 11 countries (6%) also for boys [2].

Current evidence suggests that the 3 licensed HPV vaccines have relatively similar effectiveness in preventing cervical cancer [2].

Regarding the impact of vaccination programmes at the population level, there is evidence of a reduction in high- grade cervical abnormalities among young women, and that vaccination significantly reduces the prevalence of high-risk HPV types among young women [2].

HPV vaccination programmes are also effective in reducing the incidence of anogenital warts. The quadrivalent vaccine, which includes HPV-6 and HPV-11, the HPV types that most commonly cause anogenital warts, provides high-level protection against anogenital warts in men and women and anogenital precancerous lesions in susceptible men aged 16–26 years; introduction of this vaccine was followed by a rapid decline in the prevalence of genital warts. In seronegative vaccinees, high seroconversion rates and high levels of anti-HPV antibodies against HPV-6 and HPV-11 VLPs were observed in females aged 9–45 years and in males aged 9–26 years. Vaccine efficacy studies showed that among HPV-naive individuals the quadrivalent HPV vaccine confers almost 100% protection against anogenital warts associated with HPV-6 and HPV-11, with efficacy of about 83% for all anogenital warts. When vaccinating young women regardless of their prior HPV expo- sure (having a maximum of 4 lifetime sexual partners and no history of abnormal cervical smears), efficacy against all anogenital warts was 62%. In a number of countries substantial decreases in cases of genital warts have occurred following the

introduction of a national HPV vaccination programme using quadrivalent vaccine, with reductions observed in unvaccinated young men in settings with female-only programmes indicating herd protection. In one study there was no statistical difference between the incidence of HPV types 6 and 11-related genital warts in the nonavalent vaccine and the quadrivalent vaccine cohorts. There is also some, though conflicting, evidence that the bivalent vaccine may provide some level of cross- protection against anogenital warts, although with lower efficacy than the direct protection conferred by quadrivalent vaccine [2].

For the prevention of cervical cancer, the WHO-recommended primary target population for HPV vaccination is girls aged 9–14 years, prior to becoming sexually active. Vaccination strategies should initially prioritize high coverage in this priority population. Achieving high vaccination coverage in girls (>80%) reduces the risk of HPV infection for boys.

Vaccination of secondary target populations, e.g. females aged ≥15 years or males, is recommended only if this is feasible, affordable, cost-effective, and does not divert resources from vaccination of the primary target population or from effective cervical cancer screening programmes.

In regard to cervical cancer prevention, all of the 3 licensed HPV vaccines provide high protection against HPV-16 and HPV-18, the HPV types which are associated with 71% of cervical cancer cases globally. HPV vaccines provide some cross-protection against HPV types not included in the vaccines. Based on evidence from clinical trials and post-introduction impact evaluations, the bivalent and quadrivalent HPV vaccines provide some level of cross-protection against high-risk HPV types other than 16 and 18, in particular for types 31, 33 and 45. HPV types 31, 33 and 45, the 3 types against which the bivalent and quadrivalent vaccines are reported to give cross-protection, are associated with 13% of cervical cancer cases. HPV types 31, 33, 45, 52 and 58, against which the nonavalent vaccine provides direct protection, are associated with 18% of the cases, i.e. a further 5% compared with the bivalent and quadrivalent vaccines which

confer cross- protection against HPV types 31, 33 and 45. The extent of any cross-protection against non-vaccine HPV types conferred by the nonavalent vaccine is not yet known [2].

A systematic review evaluated changes between pre- and post-vaccination periods in infection rates of high-risk HPV types other than types 16 and 18. Evidence of cross- protection was found for HPV-31 (prevalence ratio=0.73 [95% CI: 0.58–0.92]) but little evidence of cross-protection for HPV-33 and HPV-45 (prevalence ratio=1.04 [95% CI: 0.78–1.38] and 0.96 [95% CI: 0.75–1.23]).

WHO recognizes the importance of cervical cancer and other HPV-related diseases as global public health problems and reiterates the recommendation that HPV vaccines should be included in national immunization programmes. Cervical cancer, which comprises 84% of all HPV-related cancers, should remain the priority for HPV immunization. Prevention of cervical cancer is best achieved through the immunization of girls, prior to sexual debut. All 3 licensed HPV vaccines – bivalent, quadrivalent and nonavalent – have excellent safety, efficacy and effectiveness profiles [2].

Duration of protection

Antibody levels fall by about one log between the peak after the third dose and 18 months after vaccination and then level off, and have remained as high or higher than those seen after natural infection for the approximately 5 years of follow-up analysed to date. Note that the minimum protective antibody threshold for disease protection is not known. Early results from the quadrivalent vaccine trials show an increase in antibody titers to a challenge dose given five years after initial vaccination [3].

Protection against persistent infection or a combined endpoint of persistent infection and all genital diseases has been demonstrated for up to 5 years post-enrollment in phase II studies, the longest reported follow-up so far. Follow- up studies are planned for both vac- cines to determine duration of antibody and clinical protection among women through at least 14 years after dose 3 [3].

Conclusion

A relationship between HPV and cervical cancer had been established. Optimally, 100% of women who are at risk should obtain regular Pap tests. The morbidity and mortality associated with cervical cancer can be reduced. Research continues toward the development of HPV immunizations, but, at present, regular Pap tests and conscientious follow-up of abnormalities and HPV infections is the standard of care.

In developing countries, cervical cancer is the leading cause of cancer death in women, and 91% of global estimated HPV-related cancer deaths are due to cervical cancer. HPV vaccines are very effective at preventing infection and disease related to the vaccine-specific genotypes in women with no evidence of past or current HPV infection [3]. The primary target age group for HPV vaccines is likely to be pre-adolescent girls, but the cost-effectiveness of vaccinating other groups needs to be evaluated [3].

The auto-generated summarized text

HPV and Cervical Cancer

Background

HPV is the most common viral infection of the reproductive tract and is the cause of a range of conditions in both men and women, including precancerous lesions that may progress to cancer. The younger a woman becomes sexually active, the more likely she will be exposed to the virus. Persistent HPV infection is a necessary cause of cervical cancer. Persistent infections, within months or years, may progress towards premalignant glandular or squamous intra-epithelial lesions, classified histopathologically as cervical intra-epithelial neoplasia (CIN), and to cancer. co-infection with other STIs (herpes simplex, chlamydia and gonococcal infections); HPV infection is also implicated in a variable range of carcinomas

of the anus (88%), the vulva (15–48%, depending on age) and vagina (78%), the penis (51%) and the oropharynx (13–60%, depending on region). Over 90% of warts are associated with types 6 and 11. Anogenital warts can be difficult to treat and, in rare cases, can become malignant. HPV infection can occur at any age and has been re- ported in healthy young children. Among women infected with HIV, a recent meta-analysis found that almost 40% of women with no cervical cytological abnormalities had HPV infection. A normal result is the most frequent (90%–95%). A low-grade squamous intraepithelial lesion (LSIL) result is typically a result of an HPV infection in younger women (younger than 35) and in older women because of declining estrogen levels and other effects of the aging process on squamous cells. When a woman goes years between Pap tests, a precursor lesion may be missed. Excision by cone biopsy is reserved for more advanced or recurrent cases, especially cases involving disease in the endocervical canal. All of the quadrivalent vaccines, the bivalent vaccine, and the nonavalent vaccine. Using recombinant DNA technology, all 3 vaccines are prepared from the purified L1 structural protein that self-assembles to form HPV type-specific empty shells, termed virus-like particles (VLPs). Current evidence suggests that the 3 licensed HPV vaccines have relatively similar effectiveness in preventing cervical cancer. The introduction of this vaccine was followed by a rapid decline in the prevalence of genital warts. Achieving high vaccination coverage in girls (>80%) reduces the risk of HPV infection for boys. Based on evidence from clinical trials and post-introduction impact evaluations, the bivalent and quadrivalent HPV vaccines provide some level of cross-protection against high-risk HPV types other than 16 and 18, for types 31, 33 and 45. The morbidity and mortality associated with cervical cancer can be reduced.

References

1. World Health Organization. Human papillomavirus vaccines: WHO position paper, May 2017–Recommendations. *Vaccine* **2017**, *35*, 5753–5755. https://doi.org/10.1016/j.vaccine.2017.05.069.
2. Likes, W.M.; Itano, J. Human papillomavirus and cervical cancer: Not just a sexually transmitted disease. *Clin. J. Oncol. Nurs.* **2003**, *7*, 271–276. https://doi.org/10.1188/03.CJON.271-276.
3. Cutts, F.T.; Franceschi, S.; Goldie, S.; Castellsague, X.D.; De Sanjose, S.; Garnett, G.; Edmunds, W.; Claeys, P.; Goldenthal, K.; Harper, D. Human papillomavirus and HPV vaccines: A review. *Bull. World Health Organ.* **2007**, *85*, 719–726. https://doi.org/10.2471/BLT.06.038414.
